# Development and Validation of a Questionnaire Assessing the Perception and Practice of Workplace Violence Prevention Among Employers at Healthcare Facilities in North-Eastern Malaysia

**DOI:** 10.7759/cureus.34046

**Published:** 2023-01-21

**Authors:** Mohd Nizam Mohamad Yazid, Nik Rosmawati Nik Husain, Aziah Daud, Yelmizaitun Osman, Normazura Mustapa, Azlihanis Abdul Hadi

**Affiliations:** 1 Department of Community Medicine, Universiti Sains Malaysia School of Medical Sciences, Kota Bharu, MYS; 2 Occupational and Environmental Health Unit, Kelantan State Health Department, Kota Bharu, MYS; 3 Occupational and Environmental Health Unit, Melaka State Health Department, Ayer Keroh, MYS; 4 Occupational Safety and Health Unit, Ministry of Health Malaysia, Putrajaya, MYS

**Keywords:** exploratory factor analysis, questionnaire validation, employers, practice, perception, workplace violence prevention

## Abstract

Introduction

Healthcare workers have been suffering from workplace violence in alarming numbers, showing the importance of its prevention initiative. This study aims to develop and validate a new questionnaire to assess the perception and practice scores of workplace violence prevention among employers at healthcare facilities.

Methods

Existing literature has been reviewed to establish the domains and refine the items. The first drafted domain was the perception constructed by six components and 59 items. The second drafted domain was practice, consisting of six components and 41 items. Content validation was measured by a panel of experts using the item-level content validity index (I-CVI). Then, face validation analysis was carried out among 10 healthcare employers and presented as the item-level face validity index (I-FVI). Lastly, 222 participants were recruited to determine the validity and reliability of the questionnaire by using an exploratory factor analysis (EFA) and internal consistency reliability.

Results

Following content validation, two items in the practice domain were removed because of the I-CVI below 0.78. The I-CVI values of the remaining items for both domains were above 0.78, indicating good relevancy of 59 items to assess the perception and 39 items to evaluate the practice domains. The I-FVI values for both domains were above 0.80, suggesting that the participants easily understood the questionnaire. Bartlett’s test of sphericity was significant for both domains (*p*<0.001). The Kaiser-Meyer-Olkin measure was 0.879 for the perception domain and 0.941 for the practice domain. All items load above 0.6 in their respective factor. In addition, Cronbach’s alpha coefficient of reliability test ranged from 0.71 to 0.92 and from 0.82 to 0.97 for the perception and practice domains, respectively. The final revised questionnaire consisted of nine components (35 items) for perception and four components (27 items) for practice.

Conclusion

The newly developed set of questionnaires is a valid and reliable tool to assess the perception and practice of workplace violence prevention among employers at healthcare facilities.

## Introduction

Healthcare personnel work in unsafe, unsatisfactory, and unhealthy work environments at numerous healthcare facilities. A high volume of patients, crowded healthcare facilities, long waiting time, and delayed treatment may understandably anger patients or their family members. Thus, it could lead to heated discussions, quarrels, or even acts of violence between patients and healthcare workers (HCWs). Workplace violence (WPV) in healthcare facilities is unavoidable despite existing guidelines and training modules available for prevention.

WPV is an incident where workers are experiencing being abused, threatened, or attacked in conditions connected with their work, including commuting to and from work, involving either explicit or implicit challenges to their health, safety, or well-being [1]. The recognised forms of WPV are physical injury, verbal abuse, racial abuse, bullying, and sexual harassment [2]. According to different perpetrators, WPV is grouped into four types: Type I (Criminal Intent), Type II (Patient/Visitor), Type III (Worker-on-Worker), and Type IV (Organisational) [3]. Among them, Type II is the most commonly reported type of WPV against HCWs in Malaysia [4]. The WPV cases against HCWs in Malaysia are increasing yearly. The reported WPV in a public hospital was 71.3% [5] and in primary care and the community-based setting was 24.3% [6]. According to the Ministry of Health, 70% of HCWs in Malaysia experienced verbal abuse, 33% physical assault, 25% bullying, and 4% sexual harassment [4]. 

WPV carried a significant negative impact on the health sector in many ways. Violence at work is a known risk factor for depressive symptoms [7]. HCWs reportedly develop psychological illnesses such as depression and post-traumatic stress disorder [8]. In addition, WPV against HCWs has an adverse effect on their physical health such as cardiovascular disease [9], and is associated with absenteeism and high job turnover [10]. Furthermore, WPV would compromise the quality of health service provisions by impacting doctors' attitudes toward work, discouraging them from properly taking care of their patients [11]. Past research reported that medical officers refrained from executing high-risk medical interventions to avoid the fury that may occur following any unfavourable outcome [12]. Locally, the Ministry of Health, Malaysia, is experiencing an increasing trend of WPV in health facilities despite having launched and established guidelines and training modules to prevent and overcome WPV against HCWs for the past five years [13, 14]. The government has been bearing all the burdens and there is no clear solution at the moment or immediate future. 

Necessary progressive measures should be sought to overcome the issue of WPV in healthcare facilities. The healthcare industry is described as a ‘top-down organisation’ whereby employers are responsible for decision-making and instructing workers [15]. Having said that, employers, as decision-makers, are obligated to undertake measures to prevent and mitigate violence in the workplace. This highlights the importance of employers’ commitment and should be included as one of the main components in WPV prevention [16]. Unfortunately, the employers’ understanding of workplace safety, although highly required, was rather insufficient [15]. A valid assessment tool is essential to understand WPV against HCWs and to measure WPV prevention among healthcare employers. Hence, WPV prevention in healthcare facilities needs to be examined from a new perspective, particularly how healthcare employers perceive and practice it. Later information will help policymakers plan concise and targeted strategies for WPV prevention in healthcare facilities. However, the existing questionnaire focuses solely on WPV and is unsuitable for local workers. For example, the self-administered questionnaire, developed by the International Labour Office/International Council of Nurses/World Health Organisation/Public Services International on WPV in the health sector, only enables researchers to measure the incidence and form of WPV [17]. Meanwhile, the other related questionnaires primarily measure a subset of WPV, such as attitude toward aggression and job satisfaction, without a scoring scale [18,19]. Furthermore, these questionnaires have neither been translated into Malay nor adapted for local use. Therefore, this study aims to develop and validate a new questionnaire in Malay, which is culturally appropriate, to assess WPV prevention among employers at healthcare facilities in Malaysia.

## Materials and methods

Development and validation of questionnaire

**Figure 1:**
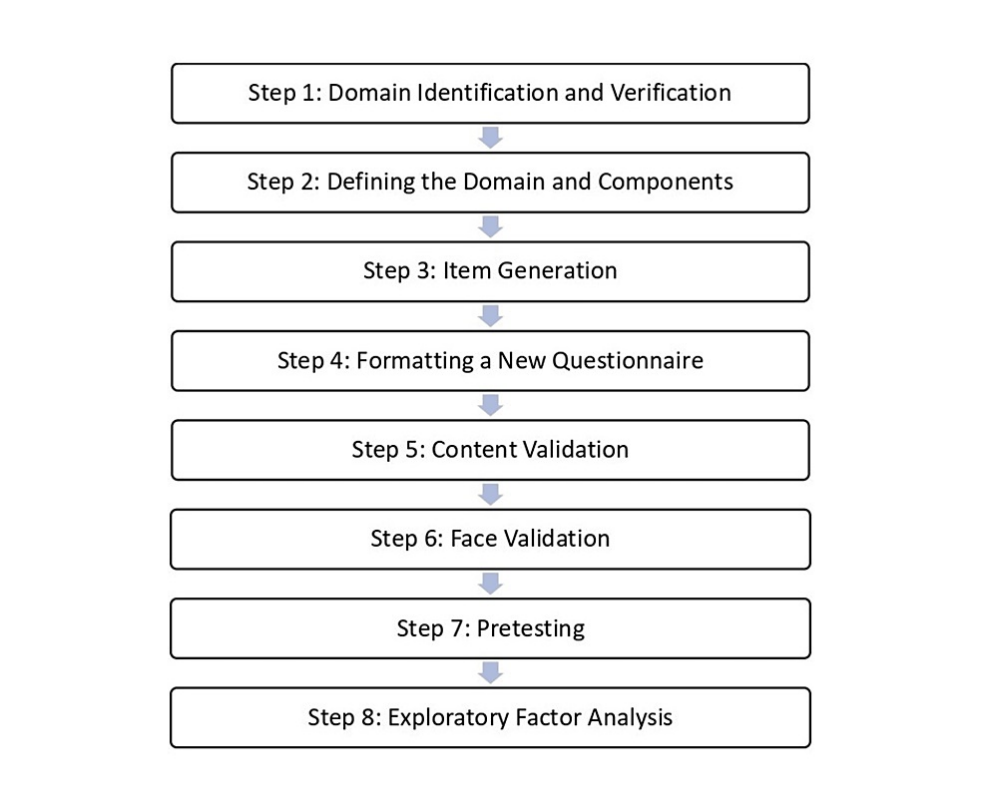
Steps of tool development and validation study

An overview of the research process begins with steps involved in developing a new questionnaire and the procedure of testing the validity and internal consistency of the instrument, as suggested in a previous study [20]. The research process can be seen in Figure 1.

Step 1: Domain Identification and Verification

The development process of the new questionnaire was initially done based on previous studies. A comprehensive literature review and group discussions with a panel of experts in WPV were conducted to determine the desired domains related to WPV prevention. The domain identification process focused on healthcare employers concerning WPV prevention. Two major domains were accepted and verified following a thorough discussion among the team members. The two domains were perception and practice, and there were no existing instruments measuring both domains that were culturally suitable for the study population.

Step 2: Defining the Domain and Components

Perception toward WPV prevention is defined as a delineation of understanding of WPV prevention topics, which individuals interpret based on prior experiences. At the same time, practice toward WPV prevention refers to a particular way of acting or responding, whether good or bad. The perception domain consists of six components: (i) form of WPV, (ii) causes of WPV, (iii) impacts of WPV, (iv) benefits of WPV prevention, (v) barriers to WPV prevention, and (vi) WPV prevention encouragement. The practice domain also contains six components: (i) workplace safety, (ii) implementation of WPV prevention, (iii) training of WPV prevention, (iv) reporting of WPV, (v) support of WPV prevention, and (vi) managerial role. 

Step 3: Item Generation

The required items for each component were then constructed. Five items per component were at least included to cover representativeness, relevancy, and consistency and were designed to convey the intended meaning. At this stage, the questionnaire contained two parts; part one consisted of proforma on general information and the other on two main domains (perception and practice). The new questionnaire comprised 13 items on the former and 100 items on the latter, which were selected as the most relevant and appropriate in the content.

Step 4: Formatting a New Questionnaire

The questionnaire was reconstructed to make it more succinct, readable, and comprehensible to participants as it became easier and quicker to fill in. For the perception domain, the six components and their respective number of items were: (i) Form of WPV (13 items), (ii) Causes of WPV (10 items), (iii) Impacts of WPV (15 items), (iv) Benefits of WPV Prevention (nine items), (v) Barriers to WPV Prevention (seven items), and (vi) WPV Prevention Encouragement (five items). For the practice domain, the six components and their respective number of items were: (i) Workplace Safety (12 items), (ii) Implementation of WPV Prevention (five items), (iii) Training of WPV Prevention (five items),(iv) Reporting of WPV (five items), (v) Support of WPV Prevention (eight items), and (vi) Managerial Role (six items), respectively. All items were close‐ended questions rated on a five‐point Likert scale ranging from 1 (strongly disagree) to 5 (strongly agree). 

Step 5: Content Validation

A panel of experts reviewed all the items to assess the relevancy and comprehensiveness of the construct items. Items with a minimum item content validation index (I-CVI) of 0.78 were considered good [21].

Step 6: Face Validation

Ten employers from healthcare facilities were appointed to review all the items by assessing the comprehensiveness and clarity of the questionnaire items. The questionnaire is considered satisfactory when the item face validation index (I-FVI) value is above 0.80 for inter-rater agreement in the questionnaire [22].

Step 7: Pretesting

Questionnaire pretesting was performed in a teaching hospital in north-eastern Malaysia, to identify any potential shortfalls in the newly developed questionnaire. The inclusion criteria include three levels of employers in healthcare facilities: director of the organisation, location supervisor, and those involved in the Occupational Safety and Health Committee (OSHC) and employers who had worked at least 12 months in the current healthcare facility. By using a convenient selection method, 30 employers were recruited to participate. Due to the coronavirus disease 2019 (COVID‐19) pandemic, the pretesting was conducted both face-to-face and online.

Step 8: Exploratory Factor Analysis (EFA)

A cross‐sectional study was conducted between August and September 2021 in north-eastern Malaysia. The study involved five categories of healthcare workplaces: hospitals, health clinics, dental clinics, district health offices, and district dental offices. The participants were employers in healthcare facilities from any of these three levels: director of the organisation, location supervisor, and those involved in the OSHC. Ministry of Health, Malaysia, has outlined the responsibility of all three different levels of employers: (i) director of the organisation, (ii) location supervisor, and (iii) OSHC for WPV prevention at healthcare facilities [14]. Workers who had worked for at least 12 months in the current healthcare facility were included. The estimated sample size was 222 participants. Initially, a list of total employers at healthcare facilities from the five categories of the workplace was gathered. Then, a stratified proportionate sampling formula was applied to determine the number of participants required for each workplace category. By using simple random sampling, participants were selected from each list of workplace categories. Due to the COVID-19 pandemic, the questionnaires were delivered online.

Data analysis was performed using IBM SPSS Statistics for Windows, Version 26.0 (Released 2019; IBM Corp., Armonk, Washington, United States) and the descriptive statistics for the questionnaire were computed. Mean scores were measured for every item and the construct validity was determined by performing the EFAs, using the principal axis factoring as the extraction method. Two EFAs were run separately as both domains had different concepts, with the first EFA for the perception domain and the second EFA for the practice domain. The Kaiser-Meyer-Olkin measure value of more than 0.7 was used to decide sample adequacy. Bartlett's test of sphericity with a p-value less than 0.05 was used as the cut-off point for data appropriateness [23]. The number of factors that constructed the domain was determined using eigenvalue >1.0 and parallel analysis using a scree plot. The construct factors were extracted using the principal axis factoring method and the item quality was assessed based on factor loading >0.6 [24]. For internal consistency, Cronbach’s alpha coefficient value of ≥0.7 was considered adequate [25].

Ethical considerations

The study was approved by these Institutional Review Boards: (i) Medical Research and Ethics Committee (MREC) of the Ministry of Health Malaysia (Approval Number: NMRR‐21‐338-57929), and (ii) the Human Research Ethics Committee of Universiti Sains Malaysia (Approval Number: USM/JEPeM/21020165). An online form with a summary of the study was created to brief the participants. The participants were first asked whether they met the inclusion criteria and consented to enrolment. The participants were then permitted to fill out the rest of the questionnaire. Data confidentiality was firmly preserved and restricted to the authors and supervisors. Afterwards, anonymous reporting and publication were conducted.

## Results

Content validation

Content validation analysis showed that I-CVI values for two items in the practice domain were below 0.78, and elimination was suggested. However, all remaining items were above 0.78, indicating good relevancy of the 59 items used to assess the perception domain and 39 items used to measure the practice domain.

Face validation

I-FVI of clarity and comprehensiveness of all items for both domains were above 0.80, indicating that the participants easily understood the questionnaire.

Pretesting

The questionnaire was generally well-received and deemed acceptable. The timing of the questionnaire distribution was adequate. The questionnaire was structured, and all the items were clear, unambiguous and easy to comprehend.

Construct validity

Characteristics of Participants

A total of 222 employers at healthcare facilities were registered for EFA; a representative from five different healthcare workplaces: hospitals (n=156, 70.3%), health clinics (n=43, 19.4%), dental clinics (n=11, 5%), district health offices (n=11, 5%), and district dental offices (n=1, 0.5%). Most participants were location supervisors (84.2%), followed by the OSHC team members (8.1%), and directors of the organisations (7.7%). Malay accounted for the majority (94.6%); 162 were women (73%), 205 (92.3%) were married, and 111 had diplomas (50%). The mean age of the participants was 44.3 years and 198 (89.2%) had more than 10 years of working experience. 

EFA and Reliability Analysis of Perception Domain

The EFA results (Table 1) provide valuable insights into the dimensionality of the latent variables. The result of Bartlett’s test of sphericity was significant with *p*<0.001. The Kaiser-Meyer-Olkin measure yielded a value of 0.879, indicating that the sample size was large enough to continue further assessment of the factor structure. The factors demonstrated sufficient convergent validity as their loadings were all above recommended threshold of 0.60 for a large sample size. As a conclusion of EFA for the perception domain, three new factors were introduced: (i) Reaction to WPV, (ii) WPV Protection, and (iii) High-Strain Job Characteristics. A total of 24 items were removed for having low factor loading. Thus, 35 items were retained in the questionnaire, which was adequate and valid.

**Table 1:** Exploratory factor analysis and reliability analysis of perception domain (n=222)

Number	Variables	Factor Loading	Communality	Cronbach’s Alpha
	Factor 1: Form of Workplace Violence			0.916
1	I believe there has been verbal intimidation in the workplace over the past year.	0.633	0.768	
2	I believe there has been physical violence in the workplace over the past year.	0.799	0.720	
3	I believe there has been an act of vandalism in my workplace over the past year.	0.777	0.683	
4	I believe there have been attempted physical assaults on my staff over the past year.	0.829	0.757	
5	I believe there has been sexual harassment in the workplace over the past year.	0.843	0.756	
6	I believe there has been an act of bullying in the workplace over the past year.	0.775	0.677	
7	I believe there has been racial harassment (racist) in the workplace over the past year.	0.656	0.592	
8	I believe there has been an act of stalking staff at work over the past year.	0.792	0.738	
	Factor 2: Benefits of Workplace Violence Prevention			0.903
9	Prevention of violence in the workplace improves the achievements of staff.	0.741	0.633	
10	Prevention of workplace violence improves the safety of staff.	0.701	0.689	
11	Prevention of workplace violence can increase staff awareness of the risk of violence incidents in the workplace.	0.762	0.697	
12	Prevention of workplace violence can reduce the cost of treatment that has to be borne due to the violent cases that occur.	0.805	0.721	
13	Prevention of violence in the workplace can reduce the cost of compensation to be incurred as a result of the violent cases that occur.	0.813	0.727	
14	Prevention of workplace violence will improve the image of the organisation.	0.819	0.738	
	Factor 3: Barriers to Workplace Violence Prevention			0.845
15	I have limited time to implement workplace violence prevention programs.	0.801	0.694	
16	I have financial constraints implementing workplace violence prevention programs.	0.795	0.725	
17	I have staff constraints to implementing workplace violence prevention programs.	0.663	0.663	
18	Staff working in remote areas is an obstacle to workplace violence prevention programs.	0.631	0.712	
19	Staff working shifts are an obstacle to workplace violence prevention programs.	0.656	0.699	
	Factor 4: Impacts of Workplace Violence			0.904
20	Violence in the workplace is an act that should not be accepted.	0.838	0.790	
21	Violence in the workplace can injure staff and damage property in the workplace.	0.855	0.827	
22	Violence in the workplace occurs due to patients or visitors failing to control their emotions or anger.	0.753	0.758	
	Factor 5: Causes of Workplace Violence			0.710
23	Violence in the workplace is an expression of a patient’s or visitor’s feelings, much like anger or growling.	0.688	0.690	
24	After committing violence in the workplace, patients or visitors feel calmer.	0.748	0.684	
25	Violence in the workplace is one of the perpetrator's methods to protect himself.	0.657	0.586	
	Factor 6: Reaction to Workplace Violence			0.727
26	Violence in the workplace is a normal reaction to feelings of anger.	0.657	0.629	
27	Violence in the workplace is a positive reaction caused by the anger of the patient or visitors while receiving treatment/running errands.	0.747	0.689	
28	Workplace violence can help staff to improve the relationship between staff and patients.	0.646	0.646	
	Factor 7: High-Strain Job Characteristics			0.847
29	Workplace violence is caused by a shortage of staff working at the scene.	0.685	0.773	
30	Workplace violence occurs due to an increase in the number of patients or the occurrence of overcrowding at work.	0.730	0.782	
31	Workplace violence prevails due to staff working in small numbers (less than 5 people).	0.658	0.727	
	Factor 8: Workplace Violence Protection			0.817
32	Workplace violence occurs due to the absence of an effective workplace violence prevention program.	0.745	0.751	
33	Workplace violence occurs due to the absence of regulation to protect staff.	0.703	0.715	
	Factor 9: Workplace Violence Prevention Encouragement			0.785
34	Increased treatment costs and workers' compensation may drive the implementation of workplace violence prevention.	0.593	0.681	
35	The time lost for patient care and work can encourage the implementation of workplace violence prevention.	0.701	0.731	

The Cronbach’s alpha for the perception domain were 0.916 for ‘Form of WPV’, 0.903 for ‘Benefits of WPV Prevention’, 0.845 for ‘Barriers to WPV Prevention’, 0.904 for ‘Impacts of WPV’, 0.710 for ‘Causes of WPV’, 0.727 for ‘Reaction to WPV’, 0.847 for ‘High-Strain Job Characteristics’, 0.817 for ‘WPV Protection’, and 0.785 for ‘WPV Prevention Encouragement’. The factors were all reflective because their indicators were highly correlated and largely interchangeable (Table 1).

EFA and Reliability Analysis of Practice Domain

The EFA results for the practice domain, constructed by 39 items, showed that Kaiser-Meyer-Olkin measure and Bartlett’s test of sphericity were 0.941 and *p*<0.001, respectively. Thus, indicating the sample size was enough to assess the factor structure. Sufficient convergent validity was demonstrated as all the factors were loaded above the recommended threshold of 0.60 for a large sample size. However, two factors were omitted from the practice domain: ‘Training of WPV Prevention’ and ‘Support of WPV Prevention’, and another 12 items were removed for having poor factor loading. Finally, 27 items were retained in the questionnaire for the practice domain, which was adequate and valid (Table 2). 

**Table 2:** Exploratory factor analysis and reliability analysis of practice domain (n=222)

Number	Variables	Factor loading	Communality	Cronbach’s Alpha
	Factor 1: Workplace Safety			0.846
1	I ensure electronic observation is provided in the workplace.	0.654	0.605	
2	I ensure security guards are provided in the workplace.	0.702	0.797	
3	I ensure that physical safety protection is provided in the workplace.	0.666	0.749	
	Factor 2: Implementation of Workplace Violence Prevention			0.967
4	I ensure workplace organisations have the power to arrest or detain individuals to be handed over to the police.	0.697	0.731	
5	I ensure that workplace organisations have the power to confiscate any weapons brought into the workplace area.	0.611	0.551	
6	I ensure that workplace organisations have mechanisms or methods to identify patients or visitors with a record of workplace violence.	0.666	0.675	
7	I ensure that workplace organisations provide additional safety arrangements (e.g. security alarms, physical barriers at workplace stations) for staff who have been victims of workplace violence.	0.720	0.710	
8	I ensure that workplace organisations have programs or policies that prevent workplace violence.	0.788	0.799	
9	I ensure that the issue of patients or visitors who are perpetrators of violence is included in the workplace violence prevention policy.	0.812	0.805	
10	I ensure that workplace organisations teach staff to report workplace violence incidents.	0.663	0.727	
11	I ensure that workplace organisations deal with violent incidents that occur outside the workplace if they are related to duty and the workplace (e.g. harassment, stalking, physical injury or verbal threats).	0.739	0.780	
12	I ensure that workplace organisations periodically review the effectiveness of workplace violence prevention programs or policies.	0.805	0.793	
13	I ensure that workplace organisations have a dedicated committee or work team that manages workplace violence prevention.	0.799	0.774	
14	I ensure workplace organisations provide staff with workplace violence prevention information materials.	0.823	0.823	
15	I ensure workplace organisations provide workplace violence prevention training to staff.	0.800	0.797	
16	I ensure workplace organisations provide separate/additional training on domestic violence prevention if needed.	0.790	0.766	
17	I ensure that staff in my organization who have experienced any incidents of workplace violence to lodge a report, including those who have not suffered injuries.	0.593	0.663	
18	I ensure that workplace violence prevention programs or policies in the organisations improve after any workplace violence incident.	0.671	0.657	
	Factor 3: Reporting of Workplace Violence			0.822
19	Over the past 12 months, incidents of workplace violence at my organisation have increased.	0.785	0.704	
20	Over the past 12 months, workplace violence incidents have affected staff in my organisation.	0.775	0.671	
21	I provide unfair service to the staff.	0.818	0.763	
22	I pay less attention to the feelings of subordinates.	0.834	0.781	
	Factor 4: Managerial Role			0.887
23	I enjoy working with my staff.	0.685	0.564	
24	I take appropriate action when bullying occurs at work.	0.762	0.750	
25	I give support to staff who experience workplace violence.	0.800	0.673	
26	I created a safe workplace environment.	0.677	0.692	
27	I accept staff opinions about workplace violence prevention programs or policies.	0.725	0.724	

The Cronbach’s alpha values for the practice domain were 0.846 for ‘Workplace Safety’, 0.967 for ‘Implementation of WPV Prevention’, 0.822 for ‘Reporting of WPV’, and 0.887 for ‘Managerial Role’. All the factors were reflective because their indicators were highly correlated and largely interchangeable as shown in Table 2.

## Discussion

This study was conducted to evaluate the psychometric properties of the perception and practice toward the WPV prevention questionnaire, starting with the development and followed by validity and reliability testing among employers at healthcare facilities in north-eastern Malaysia.

The satisfactory response rates and completeness of item responses provide evidence of the questions' relevance, intelligibility, and the questionnaire's validity [26]. The proposition to generate and select items in the questionnaire has strengthened the scale's content validity. The relevance and representativeness of each item to a particular subject were assessed by a panel of experts using the I-CVI value. Items with a minimum I-CVI of 0.78 should be retained [21]. Two items were removed from this questionnaire due to I-CVI less than the acceptable value. The questionnaire was rated on a five-point scale instead of a four-point scale to minimise selection bias [27]. The middle category can be interpreted as a neutral point, giving a person the option of being neutral and preventing forced decision-making toward a specific point. The content validation for the perception domain showed good relevancy for all the 59 items. A similar finding of content validation was observed for the 39 items in the practice domain.

Face validation is required to ensure the participants perceive the questions correctly as intended and increase the perceived relevance of the questionnaire [28]. The items are more likely to represent the construct if tested with people from the target demographic [29]. For each item, the I-FVI of clarity and comprehension of the newly developed questionnaire was above 0.80, indicating that the construct items were acceptable for inter-rater agreement in the questionnaire [22].

The EFA results using principal axis factoring as the extraction method indicated that the newly developed questionnaire had an adequate factor structure. The EFA revealed that the newly developed questionnaire contained two domains (perception and practice) with 13 components. Several items needed to be considered for being removed from the questionnaire following the analysis. Based on the principles, the removal of certain items was based on item discriminations and difficulties, the values of empirical data (factor loading), rational decision-making, and the clarity and relevance of the items relating to the guiding theoretical background [30]. Besides that, the wording of the item structure may also be considered for revision if the removed items changed the meaning of the scale. Hence, items should be removed once they were proven to have the least empirical and conceptual fit for the questionnaire as a whole. Following EFA, a total of 24 items were removed from the perception domain and 12 items were removed from the practice domain. 

Finally, the perception domain consisted of 35 items constructed in nine components: (i) form of WPV, (ii) benefits of WPV prevention, (iii) barriers to WPV prevention, (iv) impacts of WPV, (v) causes of WPV, (vi) reaction to WPV, (vii) high-strain job characteristics, (viii) WPV protection, and (ix) WPV prevention encouragement. In contrast, the practice domain consisted of 27 items constructed in four components: (i) workplace safety, (ii) implementation of WPV prevention, (iii) reporting of WPV, and (iv) managerial role. The number of items constructed for the perception and practice domains was adequate and valid as shown by the Kaiser-Meyer-Olkin measure of more than 0.7, a significant Bartlett’s test of sphericity (*p*<0.001) [23], and values of factor loading above the recommended threshold of 0.60 [28]. In addition, Cronbach’s alpha coefficient of reliability test for the perception domain of 0.71 to 0.92 and the practice domain of 0.82 to 0.97 suggested that the newly developed questionnaire was reliable. Previous studies indicated that Cronbach’s alpha of more than 0.5 was acceptable [31].

Based on the results of this cross-sectional study, a new 62-item of perception and practice toward the WPV prevention questionnaire was proposed to be used among employers at healthcare facilities. This study focused on different levels of employers at healthcare facilities on WPV prevention, which is considered the prime strength of the current research. Most existing studies only involved employees in the workplace setting and focused on the incidence of WPV. Another strength was that the newly developed questionnaire was adapted to the Malay language and the local context. Thus, it can be used as a tool for future research in similar populations and cultures. As for limitation, the number of items retained for both domains was excessive and might be burdensome to be used in the healthcare setting. Therefore, further study on confirmatory factor analysis is required to improve these questions’ quality and reduce the set of items [32]. Besides, the perception and practice toward the WPV prevention questionnaire could not be compared to the other scales available because there were none.

## Conclusions

This study shows that the perception and practice of the WPV prevention questionnaire have good psychometric properties. The validation and reliability of the questionnaire have been tested using content validation by a panel of experts, face validation by the employers at healthcare facilities, and construct validity by using EFA. Therefore, the questionnaire is valid and reliable for measuring the perception and practice toward WPV prevention among employers at healthcare facilities. The questionnaire comprises 62 items, consisting of 35 measures on the perception domain and another 27 measures on the practice domain. The items’ wording is appropriate and culturally acceptable.

## References

[1] (2022). Framework Guidelines for Addressing Workplace Violence in the Health Sector. https://www.who.int/publications/i/item/9221134466.

[2] Di Martino V, Musri M (2022). Guidance for the Prevention of Stress and Violence at the Workplace. Guidance for the Prevention of Stress and Violence at the Workplace.

[3] Bowie V, Fisher BS, Cooper C (2012). Workplace Violence. Workplace Violence.

[4] Hadi AB (2021). Bully and Harrassment in Healthcare Industry: What Are Our Roles in Prevention: Technical Update, Academy of Occupational & Environmental Medicine, Malaysia (AOEMM). https://www.aoemm.org.my/wp-content/uploads/2019/07/Bully-Harassment-in-Healthcare-Industry-What-are-Our-Roles-in-Prevention-.pdf..

[5] Zainal N, Rasdi I, Saliluddin SM (2018). The risk factors of workplace violence among healthcare workers in public hospital. Malaysian J Med Heal Sci.

[6] Ibrahim MS, Ariffin AA (2020). Workplace violence among healthcare workers in a health district and its predicting factors. Int J Public Heal Clin Sci.

[7] da Silva AT, Peres MF, Lopes Cde S, Schraiber LB, Susser E, Menezes PR (2015). Violence at work and depressive symptoms in primary health care teams: a cross-sectional study in Brazil. Soc Psychiatry Psychiatr Epidemiol.

[8] Ashton RA, Morris L, Smith I (2018). A qualitative meta-synthesis of emergency department staff experiences of violence and aggression. Int Emerg Nurs.

[9] Xu T, Magnusson Hanson LL, Lange T (2019). Workplace bullying and workplace violence as risk factors for cardiovascular disease: a multi-cohort study. Eur Heart J.

[10] Gerberich SG, Church TR, McGovern PM (2004). An epidemiological study of the magnitude and consequences of work related violence: the Minnesota Nurses' Study. Occup Environ Med.

[11] Kumari A, Kaur T, Ranjan P, Chopra S, Sarkar S, Baitha U (2020). Workplace violence against doctors: characteristics, risk factors, and mitigation strategies. J Postgrad Med.

[12] Kynoch K, Wu CJ, Chang AM (2011). Interventions for preventing and managing aggressive patients admitted to an acute hospital setting: a systematic review. Worldviews Evid Based Nurs.

[13] (2022). Guidelines for Preventing and Dealing with Violence Against Health Workers at Ministry of Health Malaysia Facilities (Document in Malay). https://jknlabuan.moh.gov.my/v4/images/gp/GP_Mencegah_Kekerasan.pdf.

[14] MOH M: Modul Latihan Mencegah & Menangani Kekerasan Terhadap Anggota Di Fasiliti Kementerian Kesihatan Malaysia. (2018 (2022). Cawangan Kwaliti Penjagaan Perubatan: Guidelines & References (Webpage in Malay). https://medicaldev.moh.gov.my/ckpp/garispanduan-rujukan/#7-26-wpfd-unit-keselamatan-kesihatan-pekerjaan-borang-1-2.

[15] Kunyk D, Craig-Broadwith M, Morris H, Diaz R, Reisdorfer E, Wang J (2016). Employers' perceptions and attitudes toward the Canadian national standard on psychological health and safety in the workplace: a qualitative study. Int J Law Psychiatry.

[16] (2020). Violence In The Emergency Care Setting. Violence In The Emergency Care Setting.

[17] (2022). Workplace Violence in the Health Sector - Country Case Study Research Instruments - Survey Questionnaire. https://www.who.int/publications/m/item/workplace-violence-in-the-health-sector---country-case-study-research-instruments---survey-questionnaire.

[18] Spector PE (1985). Measurement of human service staff satisfaction: development of the Job Satisfaction Survey. Am J Community Psychol.

[19] Palmstierna T, Barredal E (2006). Evaluation of the perception of aggression scale (POAS) in Swedish nurses. Nord J Psychiatry.

[20] Boateng GO, Neilands TB, Frongillo EA, Melgar-Quiñonez HR, Young SL (2018). Best practices for developing and validating scales for health, social, and behavioral research: a primer. Front Public Health.

[21] Lynn MR (1986). Determination and quantification of content validity. Nurs Res.

[22] Yusoff MSB (2019). ABC of content validation and content validity index calculation. Educ Med J.

[23] Hair JF, Black WC, Babin BJ, Anderson RE (2013). Multivariate Data Analysis (7th ed).

[24] Awang Z (2012). Research Methodology and Data Analysis Second Edition. Research Methodology and Data Analysis Second Edition.

[25] DeVellis RF, Thorpe CT (2022). Scale development : theory and applications. Scale Development: Theory and Applications.

[26] Bowling A (1997). Investigating Health and Health Services. Research Methods in Health: Investigating Health and Health Services.

[27] de Alwis MP, Lo Martire R, Äng BO, Garme K (2016). Development and validation of a web-based questionnaire for surveying the health and working conditions of high-performance marine craft populations. BMJ Open.

[28] Holden RR (2010). Face Validity. Corsini Encycl Psychol.

[29] Haynes SN, Richard DC, Kubany ES (1995). Content validity in psychological assessment: a functional approach to concepts and methods. Psychol Assess.

[30] Stanton JM, Sinar EF, Balzer WK, Smith PC (2002). Issues and strategies for reducing the length of self report scales. Pers Psychol.

[31] Morera OF, Stokes SM (2016). Coefficient α as a measure of test score reliability: review of 3 popular misconceptions. Am J Public Health.

[32] Streiner DL, Norman GR (2008). Health Measurement Scales: A Practical Guide to Their Development and Use (4th ed). https://academic.oup.com/book/6813.

